# Short Telomeres Compromise β-Cell Signaling and Survival

**DOI:** 10.1371/journal.pone.0017858

**Published:** 2011-03-10

**Authors:** Nini Guo, Erin M. Parry, Luo-Sheng Li, Frant Kembou, Naudia Lauder, Mehboob A. Hussain, Per-Olof Berggren, Mary Armanios

**Affiliations:** 1 Department of Molecular Biology and Genetics, Johns Hopkins University School of Medicine, Baltimore, Maryland, United States of America; 2 Medical Scientist Training Program, Johns Hopkins University School of Medicine, Baltimore, Maryland, United States of America; 3 Department of Oncology, Johns Hopkins University School of Medicine, Baltimore, Maryland, United States of America; 4 The Roft Luft Research Center for Diabetes and Endocrinology, Karolinska Institutet, Stockholm, Sweden; 5 Department of Medicine, Johns Hopkins University School of Medicine, Baltimore, Maryland, United States of America; 6 Department of Pediatrics, Johns Hopkins University School of Medicine, Baltimore, Maryland, United States of America; 7 McKusick-Nathans Institute of Genetic Medicine, Johns Hopkins University School of Medicine, Baltimore, Maryland, United States of America; Texas A&M University, United States of America

## Abstract

The genetic factors that underlie the increasing incidence of diabetes with age are poorly understood. We examined whether telomere length, which is inherited and known to shorten with age, plays a role in the age-dependent increased incidence of diabetes. We show that in mice with short telomeres, insulin secretion is impaired and leads to glucose intolerance despite the presence of an intact β-cell mass. In *ex vivo* studies, short telomeres induced cell-autonomous defects in β-cells including reduced mitochondrial membrane hyperpolarization and Ca^2+^ influx which limited insulin release. To examine the mechanism, we looked for evidence of apoptosis but found no baseline increase in β-cells with short telomeres. However, there was evidence of all the hallmarks of senescence including slower proliferation of β-cells and accumulation of p16*^INK4a^*. Specifically, we identified gene expression changes in pathways which are essential for Ca^2+^-mediated exocytosis. We also show that telomere length is additive to the damaging effect of endoplasmic reticulum stress which occurs in the late stages of type 2 diabetes. This additive effect manifests as more severe hyperglycemia in Akita mice with short telomeres which had a profound loss of β-cell mass and increased β-cell apoptosis. Our data indicate that short telomeres can affect β-cell metabolism even in the presence of intact β-cell number, thus identifying a novel mechanism of telomere-mediated disease. They implicate telomere length as a determinant of β-cell function and diabetes pathogenesis.

## Introduction

The incidence of type 2 diabetes increases with age and one in five individuals are affected by the age of 60 [1]. The natural history of type 2 diabetes is progressive. In its early stages, type 2 diabetes manifests as impaired glucose tolerance and defective insulin secretion which occur in the presence of an intact β-cell mass [2]. This is followed by a frank loss of β-cells and a concomitant need for insulin therapy [2], [3], [4]. Recent genomic studies have underscored the influence of inherited factors that affect β-cell integrity and function in age-related diabetes [5], [6], [7], [8]. While these germline factors are hypothesized to play a role in the pathogenesis of diabetes, the biology that underlies their increasing penetrance with age is not understood.

Telomeres shorten progressively with cell division, and short telomeres activate a DNA damage response that leads to apoptosis and senescence [9], [10]. Telomerase synthesizes new telomere repeats onto chromosome ends to offset in part this telomere shortening [11], [12]. Mutations in *TERT*, the telomerase reverse transcriptase, and *TR*, the telomerase RNA, cause telomere shortening and a degenerative organ failure syndrome that manifests prominently in tissues of rapid turnover: the skin, mucosa and bone marrow [13], [14]. This disease complex is often recognized in dyskeratosis congenita (DC), a premature aging syndrome defined by classic mucocutaneous features [15]. In DC and related disorders, short telomeres cause stem cell loss and progressive organ dysfunction which leads to premature mortality due to bone marrow failure and pulmonary fibrosis (reviewed in [15]). Even when the telomerase locus is wild-type, short telomeres are sufficient to cause age-associated degenerative disease similar to the DC phenotype [16], [17]. This observation has pointed to short telomere length as the relevant lesion in the setting of mutant telomerase genes, and more commonly, because telomere length is polymorphic, as an important genetic determinant of age-related disease [17].

Because β-cell function declines with age, we hypothesized that short telomeres might contribute to β-cell failure and to the increasing incidence of diabetes with age. We show, in a genetically defined model, that short telomeres are sufficient to impair glucose homeostasis. Mice with short telomeres have defects in insulin release. In *ex vivo* studies, we show short telomeres limit insulin exocytosis because of signaling defects. These include impaired mitochondrial membrane hyperpolarizaion and Ca^2+^ handling which are essential for intact insulin exocytosis. In the setting of ER stress, short telomeres compromise β-cell mass and worsen diabetes severity by inducing apoptosis. We also find a relatively increased incidence of diabetes in patients with DC who have short telomeres. Our data implicate telomere length as a critical determinant of β-cell function and diabetes risk.

## Results

### Mice with short telomeres have impaired glucose tolerance and glucose-stimulated insulin release

**Figure 1:**
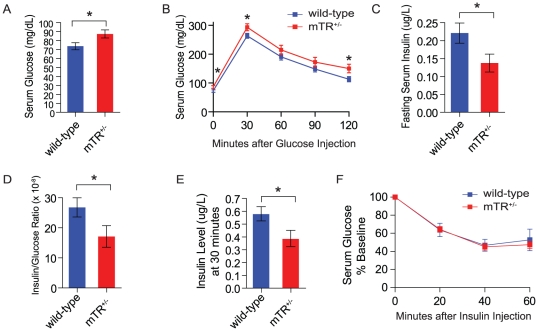
Mice with short telomeres have defective insulin secretion. **A**. mTR^+/−^ with short telomeres have fasting hyperglycemia compared with wild-type mice (n = 25/group). **B**. Higher mean serum glucose during an intraperitoneal 2 hour glucose tolerance test in mice with short telomeres (n = 25/group). **C**. mTR^+/−^ mice with short telomeres have lower fasting insulin levels. **D**. The fasting insulin is lower even when corrected for serum glucose. **E**. When challenged with glucose, mTR^+/−^ with short telomeres have lower insulin levels. **F**. Insulin tolerance test shows mTR^+/−^ mice with short telomeres have intact peripheral insulin sensitivity. Serum glucose was measured after insulin injection at the timepoints shown. Mice were 3–4 months of age. For **C–F**, n = 10–14/group. Error bars represent SEM. * indicates two-sided P-value<0.05.

To test whether short telomeres impair glucose homeostasis, we studied late generation CAST/EiJ mice that are heterozygous null for telomerase RNA, mTR^+/−^, and have short telomeres (Figure S1A). Although they were lean, mTR^+/−^ mice with short telomeres had fasting hyperglycemia compared with wild-type mice (Figure 1A and Figure S1B). When challenged in a 2 hour glucose tolerance test, mTR^+/−^ mice with short telomeres had relatively higher serum glucose levels compared with controls (Figure 1B). To determine whether the glucose intolerance was due to islet-intrinsic or extrinsic factors, we measured fasting insulin levels and found mTR^+/−^ mice with short telomeres had lower levels (Figure 1C). The insulin level was inappropriately low even after correcting for the serum glucose (Figure 1D). We next examined insulin release in response to a glucose stimulus, and found that mice with short telomeres had impaired insulin secretion (Figure 1E). There were no impairments in glucose uptake in an insulin tolerance test indicating mTR^+/−^ mice with short telomeres had no defects in peripheral insulin sensitivity (Figure 1F). To exclude a developmental defect, we examined islet architecture and histology but did not detect any abnormalities (Figure S1C–1D). Moreover, when we examined islets for insulitis, we found no infiltrates. β-cell mass quantifies the total insulin producing capacity accounting for the insulin positive area, the total pancreas section area, as well as the pancreas weight [18]. When we measured β-cell mass, we found that it was intact (Figure 1SE). The insulin content and the percent of β-cells per islet were also intact in mice with short telomeres compared with controls, and there was no change in individual β-cell size (Figure 1SF–H). The glucose intolerance, lower insulin levels, and defective glucose-stimulated insulin release were also present in a second strain of mice with short telomeres [mTR^−/−^ fourth generation (G_4_) mice] on the C57BL/6 background. In this strain, telomere-mediated phenotypes are seen only when telomerase is null, and after successive generations of breeding [10], [19] (Figure S2A–2D). The lower basal insulin levels and glucose-stimulated insulin release were not seen in early generation mTR^−/−^ G_1_ mice which have long telomeres (Figure S2E–G), indicating that the absence of telomerase alone was not sufficient to cause these defects. These data, in two independent genetic backgrounds, indicated that short telomeres impair glucose tolerance because of defective insulin release. This defect is independent of β-cell mass, size and insulin content.

### Short telomeres cause mitochondrial dysfunction and impair Ca^2+^ handling in islets

**Figure 2:**
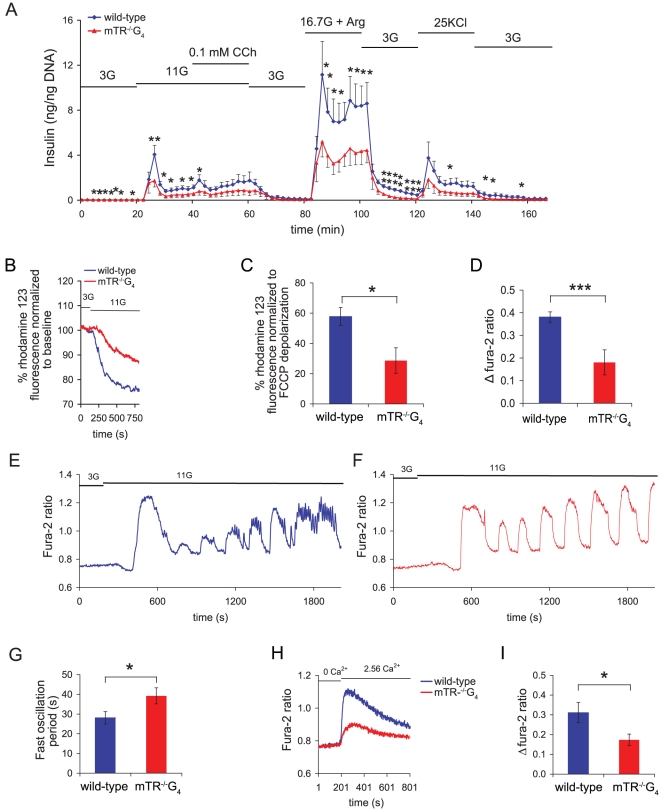
Impaired insulin release, mitochondrial function and Ca^2+^ handling in islets with short telomeres. **A**. Dynamic insulin secretion in islets from mTR^−/−^G_4_ mice compared with wild-type mice. Bars above the traces indicate the duration of stimulation. 3G, 11G, 16.7G+Arg and 25KCl indicate 3 mM and 11 mM glucose, 16.7 mM glucose plus 20 mM Arginine, and 25 mM KCl, respectively. CCh indicates carbachol. Insulin was measured every 2 minutes. Data are mean insulin level (ng/ng DNA). * Indicates P-value<0.05 and ** ≤0.01 (one-sided). **B**. Measurements of mitochondrial membrane potential in response to 11 mM glucose in islets from mTR^−/−^G_4_ mice. The rhodamine 123 fluorescence decreases when the mitochondrial membrane polarizes. **C**. Data are means of decrease in % rhodamine 123 fluorescence normalized to carbonyl cyanide 4-(trifluoro-methoxy) phenylhydrazone (FCCP) depolarization. **D**. Decreased Δ peak [Ca^2+^]_i_ values in islets from mTR^−/−^G_4_ and control mice after stimulation with 11 mM glucose. (**E**) and (**F**) Fura-2 fluorescence ratio is shown. Bars above the traces indicate the duration of stimulation. 3G and 11G indicate 3 mM and 11 mM glucose, respectively. The traces are representatives of 12–13 experiments from four islet preparations. **G**. Glucose stimulated fast [Ca^2+^]_i_ oscillation frequency in isolated islets from mTR^−/−^G_4_ and control mice. **H**. Effects of adding 2.56 mM CaCl_2_ in the perfusion chamber on changes in [Ca^2+^]_i_, indicating Ca^2+^ influx over the plasma membrane. Example tracings of Fura-2 fluorescence ratio are shown. Bars above the traces indicate the duration of stimulation. 0 Ca^2+^ and 2.56 Ca^2+^ indicate 0 mM CaCl_2_ plus 2 mM EGTA and 2.56 mM CaCl_2_, respectively. The traces are representatives of 10 experiments from four islet preparations. **I**. Means in Δfura-2 fluorescence ratio is shown for (**H**). Mice were 10 months old (wild-type n = 5, mTR^−/−^G_4_ n = 6). Error bars represent SEM. For **B–I**, *, ** and *** indicate two-sided P-value<0.05, 0.01 and 0.001, respectively.

To probe the mechanisms underlying the telomere-mediated β-cell dysfunction, we isolated islets and examined the kinetics of insulin secretion *ex vivo*. In response to glucose stimulus, islets from short telomere mice had defective insulin release *in vitro* (Figure 2A). The defects were noted in both the first and second phases of insulin release, as well as at maximum release induced by arginine (Figure 2A). Insulin secretion was also impaired when exocytosis was directly stimulated by KCl depolarization (Figure 2A). These data established that short telomeres significantly impair insulin release.

Glucose-stimulated insulin release is signaled by an increase in the ATP/ADP ratio, closure of ATP-dependent K^+^-channels, depolarization of the β-cell plasma membrane, opening of voltage dependent L-type Ca^2+^-channels and an increase in cytoplasmic free Ca^2+^ concentration ([Ca^2+^]_i_), resulting in the release of insulin containing secretory granules [20]. To define the mechanisms underlying the telomere-mediated defects in insulin secretion, we first measured mitochondrial membrane potential in isolated islets after a glucose stimulus. When the mitochondrial membrane polarizes, a change in rhodamine 123 fluorescence reflects the extent of mitochondrial membrane hyperpolarization [20]. In response to glucose, we found a decrease in membrane hyperpolarization in islets with short telomeres, consistent with a defect in the respiratory chain (Figure 2B–2C). Since Ca^2+^ is the main trigger of exocytosis, we next examined its influx in response to glucose stimulation and found an impairment in [Ca^2+^]_i_ rise in islets with short telomeres (Figure 2D). It is well-established that subsequent to glucose stimulation, [Ca^2+^]_i_ increases and decreases in an oscillatory manner, reflecting an intricate balance between Ca^2+^ influx over the plasma membrane and Ca^2+^ mobilization from intracellular stores [20]. These [Ca^2+^]_i_ oscillations are essential for appropriate β-cell function. Although islets from mice with short telomeres had intact slow [Ca^2+^]_i_ oscillations, there was a decrease in the frequency of fast [Ca^2+^]_i_ oscillations, indicating a functional defect in β-cell Ca^2+^ handling (Figure 2E–2G). When we examined Ca^2+^ influx over the plasma membrane in the absence of glucose, we also found impairments in mutant islets (Figure 2H–2I). Hence, the insulin secretion defect caused by short telomeres is multi-factorial and mediated by β-cell autonomous defects in both mitochondrial function as well as deteriorated Ca^2+^ handling.

### Short telomere islets have the hallmarks of senescence

**Figure 3:**
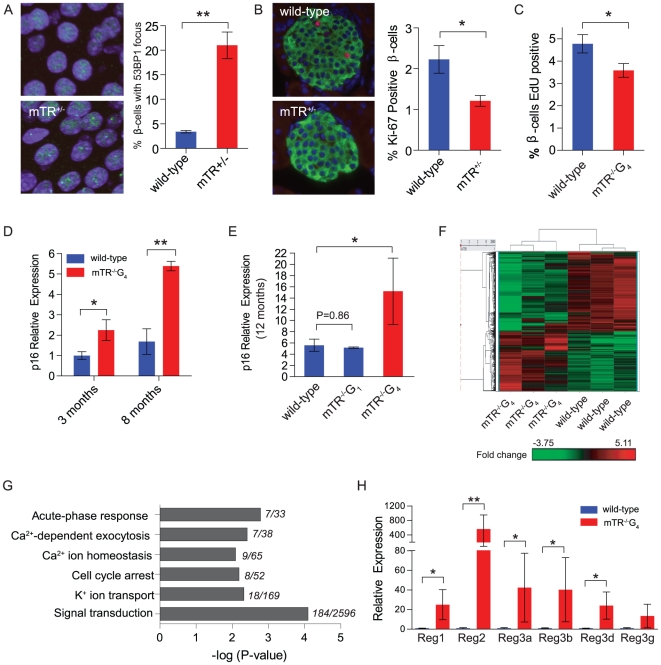
Islets from mice with short telomeres have hallmarks of senescence. **A**. DNA damage foci in β-cells from mTR^+/−^ late generation mice were detected by immunofluorescence against 53BP1 (green) in nuclei (blue). Analysis was limited to insulin positive cells and cells were deemed positive if they had at least one focus (n = 5 mice/group, 4 months old). **B**. Immunofluorescence images from wild-type and mTR^+/−^ mice with short telomeres. Proliferating β-cells are positive for both insulin (green) and Ki-67 (red) (n = 5 mice/group, 50 islets/mouse, 6 weeks old). **C**. The percent of β-cells with incorporated EdU was lower in mTR^−/−^G_4_ C57BL/6 mice after a 14 day pulse (n = 5/group, 50 islets/mouse, 6 months old). **D**. Relative expression of p16*^INK4a^* in islets shows a gradual increase with age in wild-type mice. mTR^−/−^G_4_ mice have higher levels at the age groups shown. **E**. p16*^INK4a^* expression in islets was not increased in mTR^−/−^G1 mice. For **D** and **E**, n = 3–6 mice/timepoint. **F**. Heat map of mRNA microarray data shows differential expression profiles in wild-type compared with mTR^−/−^G_4_ mice. The red color expresses genes that are up-regulated and green down-regulated genes. The fold-change based on color is shown in the key below **F**. Heat map is based on genes with 1.5-fold expression change (n = 3 mice/group, 15 months old). Values were normalized by subtracting the mean of all samples. Fold change indicated in the key is log_2_-based. **G**. β-cell relevant pathways that are altered in the gene ontology analysis plotted relative to the significance of the P-value. Only pathways with P-value<0.01 were analyzed. The number of genes altered relative to the total retrieved is shown to the right of the bar graph. **H**. qRT-PCR verification of Reg gene family expression shows significant increases in mTR^−/−^G_4_ mice (n = 4 mice/group, 15 months old). Error bars represent SEM. * indicates two-sided P-value<0.05 and ** P-value<0.01.

The defects in insulin exocytosis suggested that DNA damage associated with short telomeres might provoke global β-cell dysfunction even when β-cell mass is preserved. To test whether in β-cells with short telomeres there is evidence of DNA damage, we examined 53BP1 foci and found an increase compared with controls (Figure 3A). Short telomeres are a potent inducer of senescence, we therefore examined the hallmarks of senescence and tested whether there was evidence of impaired proliferation and accumulation of cyclin dependent kinase inhibitors. At 6 weeks of age, β-cells in mTR^+/−^ mice with short telomeres had a lower proliferative fraction as measured by Ki-67 immunostaining of insulin positive cells (Figure 3B). The slower β-cell proliferation was also detected in C57BL/6 mTR^−/−^G_4_ mice (Figure 3C). We next isolated islets and quantitated p16*^INK4a^* transcript levels by real time RT-PCR. p16*^INK4a^* levels increased in wild-type mice with age, as shown previously [21] (Figure 3D–3E). However, at every age group we examined, short telomeres induced p16*^INK4a^* prematurely and, by 8 months of age, there was a 3-fold up-regulation compared to wild-type mice (Figure 3D–3E). The accumulation of p16*^INK4a^* was a direct consequence of the short telomeres, not the absence of telomerase, as telomerase null mice with long telomeres did not show this increase (Figure 3E). The accumulation was also specific to p16*^INK4a^* and not to other genes near the *INK4a* locus (e.g. p15) or other cyclin-dependent kinase inhibitors including the p53 target p21 (Figure S3A–3C). There was a trend toward accumulation of Arf, the other *INK4a* transcript, in islets from mice with short telomeres (Figure S3D). These data indicated that short telomeres impair the cell cycle progression of β-cells and cause premature accumulation of the senescence marker p16*^INK4a^* in pancreatic islets.

### Short telomeres cause gene-expression changes in signaling pathways

We next examined the mechanism by which short telomeres may affect β-cell function in the absence of β-cell loss. Senescence is associated with altered gene expression signatures that are cell- and context-dependent [22]. We tested whether transcriptional changes in islets may explain the functional defects due to short telomeres. Microarray analysis on purified islets from wild-type and mTR^−/−^G_4_ mice showed differential expression of 1,935 genes: n = 1,153 decreased (60%) and n = 782 up-regulated. A unique transcriptional signature distinguished islets with short telomeres from controls by clustering analysis (Figure 3F). Gene ontology revealed that multiple pathways essential for insulin secretion were affected including signal transduction, Ca^2+^-mediated exocytosis, Ca^2+^ homeostasis, and K^+^ ion transport (Figure 3G and Table S1). The ontology also implicated genes involved in cell cycle control and stress response processes (Figure 3G and Table S1). The genes altered in the stress response included the Reg gene family which had the highest fold up-regulation (Figure 3H and Table S1). The Reg genes were initially identified in the regenerating pancreas, and are known to accumulate in islets from patients with type 2 diabetes [23], [24]. These data indicated that short telomeres alter islet transcriptional programs; this signature affects multiple cellular processes which are essential for insulin secretion.

### Decreased β-cell survival due to short telomeres in the setting of ER stress

**Figure 4:**
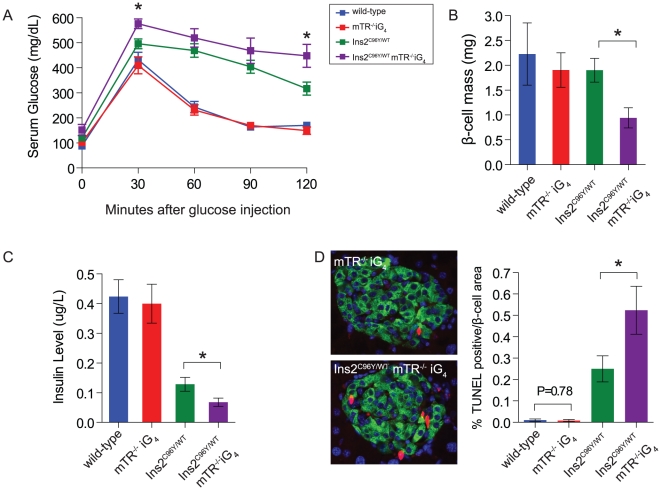
Short telomeres worsen diabetes severity in Akita mice and cause β-cell loss. **A**. Two hour glucose tolerance test of Ins2^C96Y/WT^ mTR^−/−^iG_4_ with short telomeres shows more severe impairments compared with Ins2^C96Y/WT^ mice. **B**. Ins2^C96Y/WT^mTR^−/−^iG_4_ mice have decreased β-cell mass compared with Ins2^C96Y/WT^ mice. This decrease is associated with lower basal serum insulin levels (**C**). **D**. Representative images of TUNEL (red) co-staining with insulin (green) shows an increase in β-cell apoptosis as quantitated in the bar graph. Data shown are from 8 month old females (n = 5–10 mice/group). Error bars represent SEM. * indicates two-sided P-value<0.05.

Because short telomeres cause β-cell dysfunction, we reasoned that telomere length will be a modifier of severity in monogenic forms of diabetes that affect β-cell integrity. We crossed the mTR null allele in C57BL/6 mice onto the diabetic Akita mouse that carries a mutation in the insulin gene, Ins2^C96Y/WT^, and generated double mutant mice that have short telomeres (Figure S4A). Mutations in the insulin gene cause diabetes in humans and in the Akita mouse where insulin misfolding leads to ER stress and apoptosis and clinically manifests as irreversible β-cell failure [25], [26], [27]. In Ins2^C96Y/WT^ mice, diabetes developed as expected, and was severe in males [27]. We therefore studied double mutant female mice and hypothesized that short telomeres would modulate disease severity. By 8 months, we found greater impairments in glucose tolerance in Ins2^C96Y/WT^ mice that had short telomeres (Figure 4A). The glucose intolerance was associated with a loss of β-cell mass which did not occur in Ins2^C96Y/WT^ mutant mice with long telomeres (Figure 4B). Ins2^C96Y/WT^ mice with short telomeres also had lower serum insulin levels (Figure 4C). Consistent with a β-cell intrinsic defect, these phenotypes were not due to abnormalities in insulin resistance (Figure S4B).

We next examined whether short telomeres affected the rate of β-cell apoptosis due to ER stress in Akita mice. In the absence of ER stress, the baseline cell death rate of β-cells was low, and there was no difference between wild-type and short telomere mice (Figure 4D). However, in the setting of ER stress, we identified a two-fold increase in TUNEL positive β-cells in Ins2^C96Y/WT^ mice with short telomeres compared with Ins2^C96Y/WT^ (Figure 4D). These data indicated that short telomeres cause additive β-cell dysfunction in the setting of ER stress, decreasing the threshold for apoptosis. This effect clinically manifests as more severe diabetes associated with loss of β-cell mass.

### Increased incidence of diabetes in DC

Our data indicated that short telomeres cause β-cell dysfunction in mice. To address whether telomere length contributes to disease in humans, we asked whether patients with DC might be at risk for developing diabetes. We found several published case reports that have incidentally reported on individuals who carried the diagnosis of DC and who had impaired glucose homeostasis [28], [29], [30]. These individuals were diagnosed with glucose intolerance or diabetes at 20 months, 21, 22 and 50 years of age. In our registry of families with DC and related telomere syndromes, we queried 20 consecutive individuals from 7 families, and identified 3 individuals who developed insulin-dependent diabetes prior to the age of 30. The clinical details of 2 of the 3 individuals are listed in Figure S5. Given the expected incidence of diabetes in this age group (3 per 1000), the likelihood that diabetes would occur by chance alone is low (P<0.001, chi-square test).

## Discussion

The study of the telomerase knockout mouse has particular significance for understanding the genetics of type 2 diabetes since the short telomere defect that is present in this model is acquired universally in human aging. Over the last decade, telomere-mediated phenotypes identified in the mouse have consistently converged with the human age-related disease phenotypes [15]. Here we show that short telomeres are sufficient to recapitulate the glucose intolerance and insulin secretion defects which occur in the early stages of human age-related diabetes. Telomere length is a polymorphic trait, and short telomeres are a uniquely inherited genotype that can cause degenerative disease even when telomerase is wild-type [17]. In recent cross-sectional studies, short telomeres have been associated with type 2 diabetes [31], [32], [33], [34]. Our data indicate that short telomeres are not simply associated with, but are a relevant modifier of diabetes risk and severity, and may be a valuable biomarker that identifies individuals at greatest risk in clinical settings.

In our study of mice with short telomeres, we find both *in vivo* and *ex vivo* defects in β-cell insulin secretion. The insulin secretion impairments surprisingly occur in the presence of intact insulin content and β-cell mass. The defective insulin secretion is multi-factorial and appears to be due to simultaneous, independent impairments in key exocytosis pathways including glucose-dependent mitochondrial membrane hyperpolarization, as well as glucose-independent defects in Ca^2+^ handling. Our data implicate gene expression changes in the setting of senescence as underlying these defects. The transcriptional changes we identify affect pathways essential for β-cell function including Ca^2+^-mediated exocytosis. These findings are in contrast to a recent study which reported that insulin secretion defects in late generation mTR^−/−^ mice are due to a decrease in insulin positive cells [35]. Our *ex vivo* studies indicate that short telomeres are sufficient to cause insulin secretion defects even when β-cell number is intact. Our data support a model where short telomeres induce senescence-associated gene expression changes in β-cells; this program contributes to defective signaling and clinically manifests as impaired glucose homeostasis. Short telomeres are canonically known to cause degenerative disease by inducing a loss of stem cells [10], [16]. Our findings suggest that, in some settings, even when cell mass is preserved, short telomeres compromise organ function. Our data therefore provide evidence for a novel mechanism of telomere-mediated disease where short telomeres can compromise organ homeostasis in the absence of overt cell loss.

In the Akita mouse we show that in the setting of ER stress, short telomeres cause additive injury that results in loss of β-cell mass. Short telomeres are a potent inducer of cell death [10], and in the presence of ER stress, the additive damage signals from both telomere dysfunction and the misfolded protein response evoke an apoptotic program that is otherwise not apparent in the presence of short telomeres alone. In contrast to the glucose intolerance we observed in male mTR^−/−^G_4_ mice, we did not identify baseline impaired glucose tolerance in female mTR^−/−^iG_4_. This observation is consistent with the known increased penetrance of β-cell mediated diabetes in male mice [36]. ER stress and loss of β-cell mass occur in the late stages of diabetes [2]. Our data indicate that short telomeres cooperate with ER stress to worsen diabetes severity and, because they accumulate with age, may contribute to the progressive natural history of diabetes which culminates in β-cell loss. Therefore both the senescence and apoptotic downstream consequences of telomere shortening contribute to the β-cell degenerative phenotype.

We identified an increased incidence of diabetes in a small cohort of DC patients with short telomeres. Idiopathic pulmonary fibrosis patients, who also have short telomeres, have been reported to be more prone to develop diabetes [37], [38], [39]. Diabetes incidence is known to be increased in other syndromes associated with DNA damage including Fanconi anemia where 10% of patients are affected [40]. Its association with DC, if replicated in larger cohorts, would suggest that, in addition to bone marrow failure and fibrotic disorders, diabetes may be a manifestation of telomere-mediated disease in syndromes of telomere shortening, albeit at lower penetrance. In contrast to the bone marrow failure phenotype which is prominent in the setting of short telomeres, our data indicate that telomere reserves limit the function of tissues of relatively slow turnover such as β-cells. How do we explain that short telomeres limit the function of tissues of slow turnover? One model is that although β-cell have slow turnover, they still undergo several fold expansion by adulthood [41]. Thus although the basal proliferation rate is slow, cell turnover superimposed on genetically determined shorter telomere length may be sufficient to precipitate organ dysfunction. It is also possible that, in addition, short telomere length may be a first insult that decreases the threshold for sustaining additional injury that accumulates with age such as ER stress, as our data indicate. The genetic evidence we show therefore supports that telomere function is essential for the integrity of tissues of slow turnover, and indicates that telomere shortening plays a role in a broader spectrum of specific age-related disorders than previously appreciated.

In summary, short telomeres limit insulin secretion by affecting global β-cell signaling in the setting of senescence. With ER stress, short telomeres cause increased β-cell apoptosis and loss of β-cell mass. Our data identify impaired signaling and exocytosis as a novel mechanism of telomere-mediated disease, and establish telomere shortening as a potential genetic risk factor for diabetes contributing to both its onset and pathogenesis.

## Methods

### Mouse studies

Mice were housed at the Johns Hopkins University and all procedures were approved by its Animal Care and Use Committee (approval ID MO07M492). We studied mTR^+/−^ mice on the Cast/EiJ background that were offspring of heterozygous breeding for 8–10 generations [16]. C57BL/6 mTR^−/−^ mice were bred for four generations [19]. Glucose levels were measured using a home glucometer. For glucose tolerance tests, mice were fasted overnight then injected with glucose 2.5 g/kg intraperitoneally. Serum insulin levels were measured by ELISA (Mercodia). For insulin tolerance tests, mice were injected with insulin 0.5 U/kg. To quantitate the insulin positive area, we performed immunohistochemistry on paraffin-embedded sections, 140 microns apart, and quantitated the area using Nikon Elements software. β-cell mass was determined by calculating the ratio of the insulin positive area to total pancreas sectional area, and multiplying by the pancreas weight [18]. For insulin content, whole pancreas homogenate was extracted in acid/ethanol (0.18M HCl/70% ethanol) overnight at 4°C and insulin levels were determined by ELISA after normalizing for total protein. We obtained C57BL/6 Ins2^C96Y/WT^ mice from the Jackson Laboratory. We generated Ins2^C96Y/WT^ with short telomeres using an intergenerational strategy [42]. All experiments included age-matched male mice, except in the Akita studies where we studied the more attenuated phenotype in females.

### Islet in vitro studies

Dynamic insulin release from islets was analyzed [43]. Insulin measurements were performed by a microsphere-based two-photon excitation fluorometer (TPX-technology; ArcDia Diagnostics). Insulin data were normalized to DNA content using PicoGreen (Invitrogen). Mitochondrial membrane potential was analyzed using rhodamine 123 [44]. To measure [Ca^2+^]_i_. islets were incubated with 2 µM fura-2 AM, and changes in fluorescence ratio 340/380 were analyzed [45]. Experiments were performed on a Zeiss Axiovert 200 M with a fluorescence imaging system. [Ca^2+^]_i_ oscillations were analyzed using power spectral analysis in Matlab (The Mathworks, Inc.) [46]. The amplitudes of fast and slow oscillations were calculated as the square root of the total power of periods from 6 to 60 s (FastOsc), and 60 to 600 s (SlowOsc), respectively [47]. Power spectral density for fast oscillations was calculated by the Welsh method [45], and standard fast Fourier transform power spectrum was used for slow oscillations. The dominant fast and slow periods were obtained from peaks in respective power spectrum.

### Immunofluorescence, qRT-PCR and microarray

We measured telomere length using quantitative fluorescence in situ hybridization (FISH) [37] and detected DNA damage using 53BP1 immunuofluorescence (Novus) [48]. Antibodies for insulin, glucagon, somatostatin and Ki-67 were obtained from Dako. We delivered EdU (2 mg, Invitrogen) over a 14 day period via an implanted subcutaneous pump (Alzet) and used reagents in Click-iT EdU (Invitrogen) for detection. For apoptosis studies, we performed the TUNEL assay (Roche). All histology studies were quantitated blinded to genotype. For expression studies, we purified islets [49], and extracted total RNA using RNeasy (Qiagen). To obtain sufficient RNA, we isolated islets from mTR^−/−^ mice on the C57BL/6 background which have larger islets. We performed quantitative real time RT-PCR using iQ SYBR Green Supermix (BioRad, Hercules, CA. Primer sequences are listed in Table S2. The expression of each gene was normalized to Hprt levels. Transcriptional profiling of purified islets was performed using Mouse Exon 1.0 ST Arrays (Affymetrix) at the Johns Hopkins Microarray Facility. CEL file data were extracted and normalized with Partek® Genomics Suite™ software using the Robust Multichip Analysis algorithm [50]. To explore the broadest range of transcripts, Affymetrix extended probes were imported, yielding transcripts of which 112,207 have annotation with 50,601 at the gene or mRNA-level. Genes with greater than 1.5 fold change and P-value<0.05 were considered significant. We used the Spotfire (TIBCO) platform and Gene Ontology (August 2010) to analyze gene expression. Microarray data is MIAME compliant and that the raw data has been deposited in GEO (GEO Series GSE25040). Statistical analyses were performed using GraphPad Prism software (La Jolla), and means were compared using Student's *t*-test.

### Subjects and ethics statement

Probands and family members were evaluated through the Johns Hopkins Hospital, and written informed consent was obtained from all participants. The Johns Hopkins Medicine Institutional Review Board approved the study. Individuals were eligible if they had classic mucocutaneous features of DC, or carried a mutant telomerase gene. We performed telomere length analysis using flow-cytometry and FISH, and sequenced the known DC genes from genomic DNA [37].

## Supporting Information

Figure S1 **Mice with short telomeres have intact islet morphology and β-cell mass.** **A**. Late generation mTR^+/−^ mice have short telomeres in islets as measured by quantitative fluorescence in situ hybridization. Representative images in the left panels show decreased intensity of telomere signals (red). Quantitation is shown in the bar graph (n = 5 mice/group, 15 islets/mouse, 3–4 months). **B**. mTR^+/−^ mice with short telomeres are lean (n = 21–24 mice/group, 3–4 months). **C**. Representative immunofluorescence images from wild-type and mTR^+/−^ mice with short telomeres show intact β- and α-cell histology as shown by the co-staining of insulin (green) and glucagon (red). **D**. β-cells have normal appearance and relationship to δ-cells as shown by the co-staining of insulin (green) with somatostatin (red). **E**. β-cell mass is intact in mTR^+/−^ mice with short telomeres. **F**. Total insulin content, the percentage of β-cells relative to total islet cells (**G**), and individual β-cell size (**H**) are similar in mTR^+/−^ mice with short telomeres and wild-type mice. For **G&H**, more than 1000 cells were analyzed per mouse. For **E–H**, n = 5–8 mice/group, 3–4 months of age. Error bars represent SEM. * indicates two-sided P-value<0.05 and ** P-value<0.01.
(EPS)

Figure S2 **C57BL/6 mTR^−/−^ mice with short telomeres have impaired glucose homeostasis due to defective insulin release.** **A**. Two hour intraperitoneal glucose tolerance test shows mTR^−/−^ G_4_ mice were more glucose intolerant (n = 17–20/group, 6–8 months). **B**. mTR^−/−^G_4_ mice tended to have lower fasting insulin levels, and significantly lower fasting insulin/glucose ratios (**C**). **D**. At 30 minutes after glucose injection, mTR^−/−^G_4_ mice released less insulin in response to an intraperitoneal glucose load. **E&F**. Early generation mTR^−/−^ mice (G_1_) have fasting insulin levels and fasting insulin/glucose ratio similar to wild-type controls. **G**. At 30 minutes after intraperitoneal glucose injection, mTR-/-G_1_ mice had similar insulin levels compared with controls. For **E–F**, n = 5–8/group, 10–11 months of age. Error bars represent SEM and * indicates two-sided P-value<0.05.
(EPS)

Figure S3 **Accumulation of cyclin-dependent kinase inhibitors in short telomere islets is specific to p16** ***^INK4a^*** **.** **A**. The relative expression of p15*^INK4b^* does not increase with age or with telomere shortening. **B&C**. Expression of p27 and p21 does not change drastically in pancreatic islets with age nor with short telomeres. **D**. There is a trend towards accumulation of the Arf transcript with age and with telomere shortening. 3–6 mice were analyzed for each genotype and timepoint.
(EPS)

Figure S4 **Breeding scheme for introducing the mTR^−/−^ allele and short telomeres onto the Akita (Ins2^C96Y/WT^) mouse.** **A**. Intergenerational cross of C57BL/6 Ins2^C96Y/WT^ mice with C57BL/6 mTR^+/−^ mice to generate double mutant mice was first performed. The mice were then crossed with mTR^−/−^G_3_ mouse to introduce the short telomere background. **B**. Double mutant mice have no defect in peripheral glucose uptake in this insulin tolerance test (n = 5 mice/group, 8 months old females).
(EPS)

Figure S5 **Clinical features of probands with dyskeratosis congenita and diabetes.** **A**. Clinical details of 2 patients with diabetes and dyskeratosis congenita. Case 1 had classic dyskeratosis congenita features and, in Case 2, we identified a C→G heterozygous substitution at nucleotide 204 in *hTR* which impairs telomerase activity (P<0.001, not shown). **B**. Case 1 and Case 2 have lymphocyte telomere length below the 1^st^ percentile compared with age-matched controls. Percentile lines were generated based on data from 400 controls. This range is highly predictive of a germline defect in telomerase (Alder et al *PNAS* 2008). **C**. X-linked pattern of dyskeratosis congenita inheritance in the family for index case 1. **D**. The family for index case 2 displays autosomal dominant inheritance. Both probands have relatives with a history of pulmonary and liver disease along with aplastic anemia, consistent with the diagnosis of an inherited telomere syndrome.
(EPS)

Table S1 **Biological processes and associated genes altered in microarray expression analysis of pancreatic islets from mice with short telomeres.** *Genes listed have greater than 1.5 fold expression change.
(DOC)

Table S2 **Primers used to measure expression of cyclin-dependent kinase inhibitors and Reg gene family members by qRT PCR.**
(DOC)

## References

[1] Prevention CfDCa (2008). National diabetes fact sheet: general information and national estimates on diabetes in the United States, 2007..

[2] Prentki M, Nolan CJ (2006). Islet beta cell failure in type 2 diabetes.. J Clin Invest.

[3] Butler AE, Janson J, Bonner-Weir S, Ritzel R, Rizza RA (2003). Beta-cell deficit and increased beta-cell apoptosis in humans with type 2 diabetes.. Diabetes.

[4] Yoon KH, Ko SH, Cho JH, Lee JM, Ahn YB (2003). Selective beta-cell loss and alpha-cell expansion in patients with type 2 diabetes mellitus in Korea.. J Clin Endocrinol Metab.

[5] Saxena R, Voight BF, Lyssenko V, Burtt NP, de Bakker PI (2007). Genome-wide association analysis identifies loci for type 2 diabetes and triglyceride levels.. Science.

[6] Scott LJ, Mohlke KL, Bonnycastle LL, Willer CJ, Li Y (2007). A genome-wide association study of type 2 diabetes in Finns detects multiple susceptibility variants.. Science.

[7] Zeggini E, Weedon MN, Lindgren CM, Frayling TM, Elliott KS (2007). Replication of genome-wide association signals in UK samples reveals risk loci for type 2 diabetes.. Science.

[8] Lyssenko V, Jonsson A, Almgren P, Pulizzi N, Isomaa B (2008). Clinical risk factors, DNA variants, and the development of type 2 diabetes.. N Engl J Med.

[9] Harley CB, Futcher AB, Greider CW (1990). Telomeres shorten during ageing of human fibroblasts.. Nature.

[10] Lee HW, Blasco MA, Gottlieb GJ, Horner JW, Greider CW (1998). Essential role of mouse telomerase in highly proliferative organs.. Nature.

[11] Greider CW, Blackburn EH (1989). A telomeric sequence in the RNA of Tetrahymena telomerase required for telomere repeat synthesis.. Nature.

[12] Greider CW, Blackburn EH (1987). The telomere terminal transferase of Tetrahymena is a ribonucleoprotein enzyme with two kinds of primer specificity.. Cell.

[13] Armanios M, Chen JL, Chang YP, Brodsky RA, Hawkins A (2005). Haploinsufficiency of telomerase reverse transcriptase leads to anticipation in autosomal dominant dyskeratosis congenita.. Proc Natl Acad Sci U S A.

[14] Vulliamy T, Marrone A, Goldman F, Dearlove A, Bessler M (2001). The RNA component of telomerase is mutated in autosomal dominant dyskeratosis congenita.. Nature.

[15] Armanios M (2009). Syndromes of telomere shortening.. Annu Rev Genomics Hum Genet.

[16] Hao LY, Armanios M, Strong MA, Karim B, Feldser DM (2005). Short telomeres, even in the presence of telomerase, limit tissue renewal capacity.. Cell.

[17] Armanios M, Alder JK, Parry EM, Karim B, Strong MA (2009). Short telomeres are sufficient to cause the degenerative defects associated with aging.. Am J Hum Genet.

[18] Xu G, Stoffers DA, Habener JF, Bonner-Weir S (1999). Exendin-4 stimulates both beta-cell replication and neogenesis, resulting in increased beta-cell mass and improved glucose tolerance in diabetic rats.. Diabetes.

[19] Blasco MA, Lee HW, Hande MP, Samper E, Lansdorp PM (1997). Telomere shortening and tumor formation by mouse cells lacking telomerase RNA.. Cell.

[20] Yang SN, Berggren PO (2006). The role of voltage-gated calcium channels in pancreatic beta-cell physiology and pathophysiology.. Endocr Rev.

[21] Krishnamurthy J, Ramsey MR, Ligon KL, Torrice C, Koh A (2006). p16INK4a induces an age-dependent decline in islet regenerative potential.. Nature.

[22] Zhang H, Pan KH, Cohen SN (2003). Senescence-specific gene expression fingerprints reveal cell-type-dependent physical clustering of up-regulated chromosomal loci.. Proc Natl Acad Sci U S A.

[23] Terazono K, Yamamoto H, Takasawa S, Shiga K, Yonemura Y (1988). A novel gene activated in regenerating islets.. J Biol Chem.

[24] Marselli L, Thorne J, Dahiya S, Sgroi DC, Sharma A Gene expression profiles of Beta-cell enriched tissue obtained by laser capture microdissection from subjects with type 2 diabetes.. PLoS ONE.

[25] Stoy J, Edghill EL, Flanagan SE, Ye H, Paz VP (2007). Insulin gene mutations as a cause of permanent neonatal diabetes.. Proc Natl Acad Sci U S A.

[26] Wang J, Takeuchi T, Tanaka S, Kubo SK, Kayo T (1999). A mutation in the insulin 2 gene induces diabetes with severe pancreatic beta-cell dysfunction in the Mody mouse.. J Clin Invest.

[27] Oyadomari S, Koizumi A, Takeda K, Gotoh T, Akira S (2002). Targeted disruption of the Chop gene delays endoplasmic reticulum stress-mediated diabetes.. J Clin Invest.

[28] Steier W, Van Voolen GA, Selmanowitz VJ (1972). Dyskeratosis congenita: relationship to Fanconi's anemia.. Blood.

[29] Reichel M, Grix AC, Isseroff RR (1992). Dyskeratosis congenita associated with elevated fetal hemoglobin, X-linked ocular albinism, and juvenile-onset diabetes mellitus.. Pediatr Dermatol.

[30] Walne AJ, Vulliamy T, Marrone A, Beswick R, Kirwan M (2007). Genetic heterogeneity in autosomal recessive dyskeratosis congenita with one subtype due to mutations in the telomerase-associated protein NOP10.. Hum Mol Genet.

[31] Adaikalakoteswari A, Balasubramanyam M, Mohan V (2005). Telomere shortening occurs in Asian Indian Type 2 diabetic patients.. Diabet Med.

[32] Salpea KD, Talmud PJ, Cooper JA, Maubaret CG, Stephens JW (2009). Association of telomere length with type 2 diabetes, oxidative stress and UCP2 gene variation.. Atherosclerosis.

[33] Olivieri F, Lorenzi M, Antonicelli R, Testa R, Sirolla C (2009). Leukocyte telomere shortening in elderly Type2DM patients with previous myocardial infarction.. Atherosclerosis.

[34] Sampson MJ, Winterbone MS, Hughes JC, Dozio N, Hughes DA (2006). Monocyte telomere shortening and oxidative DNA damage in type 2 diabetes.. Diabetes Care.

[35] Kuhlow D, Florian S, von Figura G, Weimer S, Schulz N Telomerase deficiency impairs glucose metabolism and insulin secretion.. Aging (Albany NY).

[36] Le May C, Chu K, Hu M, Ortega CS, Simpson ER (2006). Estrogens protect pancreatic beta-cells from apoptosis and prevent insulin-deficient diabetes mellitus in mice.. Proc Natl Acad Sci U S A.

[37] Alder JK, Chen JJ, Lancaster L, Danoff S, Su SC (2008). Short telomeres are a risk factor for idiopathic pulmonary fibrosis.. Proc Natl Acad Sci U S A.

[38] Gribbin J, Hubbard R, Smith C (2009). Role of diabetes mellitus and gastro-oesophageal reflux in the aetiology of idiopathic pulmonary fibrosis.. Respir Med.

[39] Cronkhite JT, Xing C, Raghu G, Chin KM, Torres F (2008). Telomere shortening in familial and sporadic pulmonary fibrosis.. Am J Respir Crit Care Med.

[40] Elder DA, D'Alessio DA, Eyal O, Mueller R, Smith FO (2008). Abnormalities in glucose tolerance are common in children with fanconi anemia and associated with impaired insulin secretion.. Pediatr Blood Cancer.

[41] Dor Y, Brown J, Martinez OI, Melton DA (2004). Adult pancreatic beta-cells are formed by self-duplication rather than stem-cell differentiation.. Nature.

[42] Hemann MT, Strong MA, Hao LY, Greider CW (2001). The shortest telomere, not average telomere length, is critical for cell viability and chromosome stability.. Cell.

[43] Healy JA, Nilsson KR, Hohmeier HE, Berglund J, Davis J Cholinergic augmentation of insulin release requires ankyrin-B.. Sci Signal.

[44] Silva JP, Kohler M, Graff C, Oldfors A, Magnuson MA (2000). Impaired insulin secretion and beta-cell loss in tissue-specific knockout mice with mitochondrial diabetes.. Nat Genet.

[45] Berggren PO, Yang SN, Murakami M, Efanov AM, Uhles S (2004). Removal of Ca2+ channel beta3 subunit enhances Ca2+ oscillation frequency and insulin exocytosis.. Cell.

[46] Uhlen P (2004). Spectral analysis of calcium oscillations.. Sci STKE.

[47] Stein KM, Borer JS, Hochreiter C, Okin PM, Herrold EM (1993). Prognostic value and physiological correlates of heart rate variability in chronic severe mitral regurgitation.. Circulation.

[48] d'Adda di Fagagna F, Reaper PM, Clay-Farrace L, Fiegler H, Carr P (2003). A DNA damage checkpoint response in telomere-initiated senescence.. Nature.

[49] Li DS, Yuan YH, Tu HJ, Liang QL, Dai LJ (2009). A protocol for islet isolation from mouse pancreas.. Nat Protoc.

[50] Irizarry RA, Bolstad BM, Collin F, Cope LM, Hobbs B (2003). Summaries of Affymetrix GeneChip probe level data.. Nucleic Acids Res.

