# Supplementary material for: Short Telomeres Compromise β-Cell Signaling and Survival
Source: PLoS One. 2011 Mar 10;6(3):e17858. doi: 10.1371/journal.pone.0017858 (PMC3053388; doi:10.1371/journal.pone.0017858)
Supplement: Table S1 — Biological processes and associated genes altered in microarray expression analysis of pancreatic islets from mice with short telomeres. *Genes listed have greater than 1.5 fold expression change. (DOC) [file pone.0017858.s006.doc]

| **Biological process** | **Gene symbol** | **Fold change*** | **Gene Name** |
| --- | --- | --- | --- |
| Acute phase response | Reg3b | 5.77 | regenerating islet-derived 3 beta |
|  | Reg3a | 2.87 | regenerating islet-derived 3 alpha |
|  | Serpina3n | 2.38 | serine (or cysteine) peptidase inhibitor, clade A, member 3N |
|  | Reg3g | 1.91 | regenerating islet-derived 3 gamma |
|  | Mrgpra3 | 1.89 | MAS-related GPR, member A3 |
|  | Ptger3 | 1.58 | prostaglandin E receptor 3 (subtype EP3) |
|  | Tsc2 | 1.57 | tuberous sclerosis 2 |
| Calcium-dependent exocytosis | Doc2b | 1.64 | double C2, beta |
|  | Plcd4 | 1.56 | phospholipase C, delta 4 |
|  | Tnp2 | 1.53 | transition protein 2 |
|  | Glrb | -1.50 | glycine receptor, beta subunit |
|  | Rims2 | -1.52 | regulating synaptic membrane exocytosis 2 |
|  | Rapgef4 | -1.53 | Rap guanine nucleotide exchange factor (GEF) 4 |
|  | Cacna1c | -1.62 | calcium channel, voltage-dependent, L type, alpha 1C subunit |
| Calcium ion homeostasis | Prkca | -1.71 | protein kinase C, alpha |
|  | Slc8a1 | -1.84 | solute carrier family 8 (sodium/calcium exchanger), member 1 |
|  | Slc24a2 | -1.96 | solute carrier family 24 (sodium/potassium/calcium exchanger), member 2 |
| Cell cycle arrest | Ddit3 | 1.52 | DNA-damage inducible transcript 3 |
|  | Trp53inp1 | 1.52 | transformation related protein 53 inducible nuclear protein 1 |
|  | Pkd2 | -1.52 | polycystic kidney disease 2 |
|  | Dst | -1.58 | dystonin |
|  | Mll5 | -1.64 | myeloid/lymphoid or mixed-lineage leukemia 5 |
|  | Macf1 | -1.71 | microtubule-actin crosslinking factor 1 |
|  | Ern1 | -1.75 | endoplasmic reticulum (ER) to nucleus signalling 1 |
| Potassium ion transport | Kcnip2 | -1.65 | Kv channel-interacting protein 2 |
|  | Kcnmb1 | -1.66 | potassium large conductance calcium-activated channel, subfamily M, beta member 1 |
|  | Kcnh6 | -1.67 | potassium voltage-gated channel, subfamily H (eag-related), member 6 |
|  | Hcn3 | -1.68 | hyperpolarization-activated, cyclic nucleotide-gated K+ 3 |
|  | Scn3a | -1.85 | sodium channel, voltage-gated, type III, alpha |
|  | Kcnh5 | -2.31 | potassium voltage-gated channel, subfamily H (eag-related), member 5 |
|  | Kcnip1 | -2.36 | Kv channel-interacting protein 1 |
| Signal transduction | Cckar | 3.90 | cholecystokinin A receptor |
|  | Prlhr | 2.75 | prolactin releasing hormone receptor |
|  | Sstr2 | 1.89 | somatostatin receptor 2 |
|  | Ddit4 | 1.74 | DNA-damage-inducible transcript 4 |
|  | Pde4d | -1.66 | phosphodiesterase 4D, cAMP specific |
|  | Arhgap1 | -2.00 | Rho GTPase activating protein 1 |
|  | Gng12 | -2.16 | guanine nucleotide binding protein (G protein), gamma 12 |

**Table S1. Biological processes and associated genes altered in microarray expression analysis of pancreatic islets from mice with short telomeres**

*Genes listed have greater than 1.5 fold expression change
