# Supplementary material for: Short Telomeres Compromise β-Cell Signaling and Survival
Source: PLoS One. 2011 Mar 10;6(3):e17858. doi: 10.1371/journal.pone.0017858 (PMC3053388; doi:10.1371/journal.pone.0017858)
Supplement: Table S2 — Primers used to measure expression of cyclin-dependent kinase inhibitors and Reg gene family members by qRT PCR. (DOC) [file pone.0017858.s007.doc]

**Table S2. Primers used to measure expression of cyclin-dependent kinase inhibitors and Reg gene family members by qRT PCR**

| **Name** | **Primer sequence 5’→3’** |
| --- | --- |
| p16 forward | CGGTCGTACCCCGATTCAG |
| p16 reverse | GCACCGTAGTTGAGCAGAAGAG |
| Arf forward | TGAGGCTAGAGAGGATCTTGAGAAG |
| Arf reverse | GTGAACGTTGCCCATCATCATC |
| p15 forward | AAGGACCATTTCTGCCACAG |
| p15 reverse | GCGCTGCCCATCATCATGA |
| p21 forward | GTACTTCCTCTGCCCTGCTG |
| p21 reverse | TCTGCGCTTGGAGTGATAGA |
| p27 forward | TTGGGTCTCAGGCAAACTCT |
| p27 reverse | TCTGTTCTGTTGGCCCTTTTG |
| Reg1 forward | CCAGAAGGTTCCAATGCCTA |
| Reg1 reverse | GGCGTCTGTAGTGCCACTCT |
| Reg2 forward | CCTTCCTTCTGCCAAAATCA |
| Reg2 reverse | GACCTGCATTCATGTTCTGG |
| Reg3a forward | TTATCGCTCCCACTGCTATG |
| Reg3a reverse | AAGGAAGCCTCACCTCCACT |
| Reg3b forward | CGCTGAGGCTTCATTCTTGT |
| Reg3b reverse | TGACAAGCTGCCACAGAAAG |
| Reg3d forward | CACCTGGCATTTCTGCTCAC |
| Reg3d reverse | CTGCTCCACTTCCATCCATT |
| Reg3g forward | TTCCTTCCTGTCCTCCATGA |
| Reg3g reverse | CACTCCCATCCACCTCTGTTG |
